# A Rare Case of Small Bowel Volvulus with Chylous Ascites Presumed to Be Caused by Multiple Congenital Adhesions in a Virgin Abdomen: A Case Report and Literature Review

**DOI:** 10.70352/scrj.cr.26-0017

**Published:** 2026-04-11

**Authors:** Takahiko Omameuda, Atsushi Miki, Mariko Takami, Kasumi Ogihara, Makiko Tahara, Hiroyuki Kitabayashi, Mikio Shiozawa, Masaru Koizumi

**Affiliations:** Department of Surgery, Tochigi Medical Center Shimotsuga, Tochigi, Tochigi, Japan

**Keywords:** chylous ascites, congenital adhesions, small bowel volvulus

## Abstract

**INTRODUCTION:**

Chylous ascites is an uncommon condition resulting from triglyceride-rich lymphatic fluid leakage into the peritoneal cavity and is typically diagnosed when the ascitic triglyceride level is ≥200 mg/dL. Small bowel volvulus is an uncommon surgical emergency, and its coexistence with chylous ascites is particularly rare. Postoperative adhesions are a common cause of small bowel volvulus; however, small bowel volvulus caused by adhesions presumed to be congenital in a virgin abdomen is infrequently reported. Herein, we report a case of small bowel volvulus associated with chylous ascites in which the adhesions were most consistent with congenital origin, although definitive differentiation was not possible.

**CASE PRESENTATION:**

A 61-year-old male with no history of abdominal surgery presented with sudden-onset abdominal pain. Contrast-enhanced CT revealed a whirl sign in the mesentery with mild ascites. Emergency exploratory laparotomy revealed milky-white ascites and 360° volvulus at the root of the small bowel mesentery. Multiple adhesions, presumed to be congenital based on the absence of prior surgery or documented inflammation, were identified as the underlying cause of the volvulus. All adhesions were carefully divided; de-torsion alone was sufficient, and bowel resection was unnecessary. Biochemical analysis of the ascitic fluid revealed a triglyceride level of 200 mg/dL, which confirmed the diagnosis of chylous ascites. The patient recovered uneventfully and remained recurrence-free for 6 months.

**CONCLUSIONS:**

This report describes a rare case of small bowel volvulus accompanied by chylous ascites presumed to be caused by multiple congenital adhesions in a virgin abdomen. Although causal relationships cannot be definitively established, this case highlights important considerations regarding intraoperative assessment of bowel viability and adhesions. The presence of chylous ascites alone should not be regarded as a reliable indicator of bowel ischemia; careful surgical evaluation remains essential.

## INTRODUCTION

Chylous ascites arises from the leakage of chyle, a milky fluid rich in triglycerides, into the peritoneal cavity^1,2)^; an ascitic triglyceride level ≥200 mg/dL is thus widely used as a diagnostic criterion.^3)^ Chylous ascites typically results from lymphatic vessel injury due to blunt abdominal trauma, prior abdominal surgery, or lymphatic obstruction caused by malignancy or torsion.^4)^ Meanwhile, small bowel volvulus is a rare surgical emergency, accounting for approximately 1% of all small bowel obstructions.^5)^ Coexisting chylous ascites is also uncommon. Although postoperative adhesions are a well-known cause of small bowel volvulus, congenital adhesions in a virgin abdomen, an abdomen that has not undergone prior abdominal surgery, are rarely implicated. However, it may be challenging to distinguish congenital from acquired adhesions in adult patients. Herein, we report a rare case of small bowel volvulus associated with chylous ascites, in which the adhesions are considered most consistent with congenital origin based on clinical history and intraoperative findings.

## CASE PRESENTATION

**Fig. 1:**
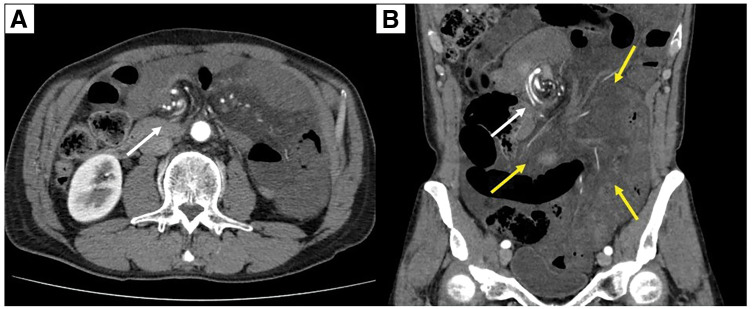
Preoperative imaging findings. (**A**) Axial section. (**B**) Coronal section. Contrast-enhanced abdominal CT demonstrates the whirl sign of a twisted mesentery (white arrows) with mild ascites. Diffuse mesenteric fat stranding is also noted (yellow arrows), with a whirl-like rotation of the mesentery and mesenteric veins around the superior mesenteric artery.

A 61-year-old male with no significant medical history experienced abdominal pain shortly after lunch. The patient appeared pale and presented to our hospital for further evaluation. On arrival, we observed no evidence of shock, and the patient’s vital signs were as follows: pulse 90 bpm, blood pressure 167/80 mmHg, and temperature 36.6°C. Laboratory investigations were largely unremarkable, with a C-reactive protein level of 0.01 mg/L, white blood cell count of 8500/μL, and hemoglobin level of 13.7 g/dL; however, the serum lactate level was elevated, at 3.0 mmol/L. Contrast-enhanced abdominal CT revealed the whirl sign of a twisted mesentery with mild ascites (**Fig. 1**). Given the high suspicion of small bowel volvulus, the patient was urgently moved to the operating room for exploratory laparotomy.

**Fig. 2:**
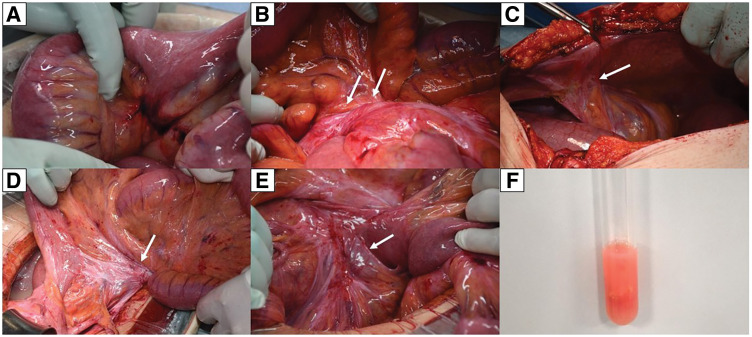
Intraoperative findings. (**A**, **B**) The small bowel mesentery appears congested and white, with a 360° rotation at the root of the small bowel mesentery (white arrows). Presumed congenital adhesions are identified intraoperatively (white arrows). (**C**) A membranous adhesion between the ascending mesocolon and the abdominal wall is indicated by a white arrow. (**D**) A white arrow indicates a dense band-like adhesion between adjacent segments of the small bowel mesentery. (**E**) A white arrow highlights a mildly dense membranous adhesion between the root of the small bowel mesentery and the sigmoid mesocolon. (**F**) Biochemical analysis of the milky-white fluid reveals a triglyceride level of 200 mg/dL.

Upon viewing the peritoneal cavity, a small volume of milky-white fluid was observed. The small bowel was distended and the mesentery was congested and white (**Fig. 2A**), with a 360° rotation at the root of the mesentery (**Fig. 2B**). Furthermore, multiple adhesions were identified within the abdominal cavity. A membranous adhesion was observed between the ascending mesocolon and abdominal wall (**Fig. 2C**), a dense band-like adhesion was identified between segments of the small bowel mesentery (**Fig. 2D**), and a moderately dense membranous adhesion was present near the base of the small bowel mesentery and sigmoid mesocolon (**Fig. 2E**). In the absence of prior abdominal surgery or documented intra-abdominal inflammation, these adhesions were considered most consistent with a congenital origin; however, definitive differentiation from acquired or subclinical inflammatory adhesions was not possible. These adhesions were considered the most likely mechanical factor contributing to small bowel volvulus and were meticulously separated to completely correct the torsion. The entire intestinal segment appeared viable following detorsion; thus, bowel resection was not required. Biochemical analysis of the fluid revealed a triglyceride level of 200 mg/dL, meeting the diagnostic criteria for chylous ascites, along with sterile cultures, supporting the diagnosis of chylous ascites (**Fig. 2F**). On POD 3, the patient developed abdominal distension and was diagnosed with paralytic ileus. Contrast-enhanced CT was performed to exclude recurrent volvulus or mechanical obstruction, and there was no evidence of recurrent torsion or bowel ischemia. The ileus resolved with conservative management, and the patient was discharged on POD 16. At the 6-month follow-up, no recurrence was observed.

## DISCUSSION

This case involved small bowel volvulus accompanied by chylous ascites in a virgin abdomen. The adhesions observed intraoperatively were presumed to be congenital; however, subclinical inflammatory processes or unrecognized peritonitis could not be completely excluded. A review of previously reported cases of this condition highlighted two key clinical insights.

First, the presence of chylous ascites did not necessarily indicate serious intraoperative complications. Chylous ascites develops via several mechanisms, including 1) direct injury to the thoracic duct or other lymphatic channels, either as a complication of abdominal surgery or as a result of traumatic abdominal injury; 2) the obstruction of lymphatic channels, as observed in malignancies (particularly lymphoma) or in intestinal mesenteric volvulus; 3) infection, most commonly peritoneal tuberculosis or filariasis; 4) congenital abnormalities of the lymphatic system; and 5) cirrhosis, which increases hepatic lymph production.^4,6,7)^

Lymphatic channels converge toward the root of the mesentery and run along the superior mesenteric artery. In small bowel volvulus, chylous ascites results from lymphatic fluid leakage into the abdominal cavity due to obstruction of these low-pressure lymphatic vessels.^8)^ As lymphatic vessels operate under lower pressure than the arterial system, they may become obstructed earlier during mesenteric torsion^3,8)^; however, the precise temporal relationship between lymphatic obstruction and arterial compromise has not been definitively established. We systematically searched the PubMed database (National Center for Biotechnology Information, National Institutes of Health, Bethesda, MD, USA) using the keywords “adult,” “chyloperitoneum,” “chylous ascites,” and “small bowel volvulus” to identify articles published between 1980 and November 30, 2025. Including our case, a total of nine small bowel volvulus cases accompanied by chylous ascites in a virgin abdomen had been reported (**Table 1**).^3,9–15)^ Among these, only one required bowel resection; this may suggest that intestinal viability is often preserved. However, we also emphasize that the small number of reported cases, heterogeneity in clinical presentation, and potential publication bias limit definitive conclusions regarding whether previously reported favorable outcomes reflect early surgical intervention, rather than a protective implication of chylous ascites itself. Therefore, the absence of bowel resection in most reported cases should be interpreted cautiously and should not preclude careful intraoperative assessment of bowel viability. In our case, volvulus detorsion alone was sufficient to complete the surgery, thus avoiding bowel resection.

**Table 1:** Small bowel volvulus with chylous ascites in a virgin abdomen: reported cases and present case

No.	Author	Age, Gender	CT findings	Bowel Resection	Degree of rotation (°)	Triglycerides (mg/dL)	Adhesion	Surgical details	Recurrence
1	Tewari N^9)^	38, M	Whirl sign	No	N/A	N/A	No	Washed out	No
2	Koh Y^10)^	19, M	Whirl sign	No	N/A	504	Coloduodenal adhesion	Detorsion + Band resection	No
3	Hayama T^11)^	70, M	Whirl sign	No	180	332	No	Detorsion	N/A
4	Leaning M^12)^	79, M	Whirl sign	No	N/A	770	Adhesions near the base of the mesentery	Detorsion	No
5	Gupta S^13)^	32, M	Internal hernia	No	N/A	360	N/A	Detorsion	No
6	Galvão D^14)^	22, M	Whirl sign	No	180	641	No	Detorsion	N/A
7	Nakamura S^3)^	93, M	Whirl sign	Partial resection of small bowel	360	670	N/A	Detorsion + Small bowel resection	N/A
8	Sinicropi T^15)^	83, M	Whirl sign	No	N/A	2488	N/A	Detorsion	N/A
9	Our case	61, M	Whirl sign	No	360	200	Multiple adhesions	Detorsion	No

M, male; N/A, not available

Second, the underlying cause of small bowel volvulus in our case consisted of adhesions presumed to be congenital in a virgin abdomen. Small bowel volvulus can be classified as primary (occurring without an underlying disease or anatomical abnormality) or secondary.^16)^ Although the mechanisms of primary small bowel volvulus remain poorly understood, contributing factors may include hypermobility of the small bowel and mesentery. Secondary small bowel volvulus occurs due to postoperative adhesions, tumors, diverticular disease, and bands.^17)^ In our case, no factors other than these presumed congenital adhesions were identified; thus, these adhesions were considered the most likely mechanical cause of the volvulus.

Although adhesions typically develop after surgery or inflammation, congenital adhesions, particularly multiple such adhesions, are rare. However, in adult patients, it is often difficult to differentiate between congenital and acquired adhesions in the absence of histopathological confirmation. In addition, congenital adhesion diagnosis in adult patients is inherently presumptive. In our case, histopathological evaluation was not performed because the adhesions were not submitted for pathological examination. This assumption was based on the absence of prior abdominal surgery or documented intra-abdominal inflammation, together with the intraoperative findings. As shown in **Table 1**, similar cases have been reported previously; therefore, this case does not establish a new clinical entity, but adds further observational data to the limited existing literature.

Furthermore, it is difficult to identify congenital adhesions on preoperative imaging. In our case, the adhesions were recognized intraoperatively and carefully released as much as possible. After adhesiolysis and detorsion, no apparent anatomical abnormality predisposing to recurrent torsion was identified, and additional fixation procedures were not performed. Fixation procedures should be considered on a case-by-case basis, depending on intraoperative findings and the perceived risk of recurrence. Surgeons should be aware that adhesions, including those presumed to be congenital, could be a potential cause of small bowel volvulus even in patients without prior abdominal surgery, and should thoroughly evaluate the abdominal cavity during surgery to identify and release any adhesions potentially responsible for the volvulus.

## CONCLUSIONS

We report an exceedingly rare case of small bowel volvulus associated with chylous ascites presumed to be caused by multiple congenital adhesions in a virgin abdomen. This case provides two important clinical insights. First, the presence of chylous ascites in small bowel volvulus should be interpreted cautiously, as its relationship with intestinal ischemia remains unclear. Second, adhesions, including those presumed to be congenital, may be an underlying cause of small bowel volvulus even in patients with a virgin abdomen. Although limited by the inherent constraints of a single case report, this study underscores the importance of cautious interpretation of chylous ascites and thorough intraoperative evaluation. Further accumulation of cases is necessary to better understand the clinical implications of this rare presentation.

## DECLARATIONS

### Availability of data and materials

All data supporting the conclusions of this study are included in this published article.
